# Mathematical Modelling to Guide Drug Development for Malaria Elimination

**DOI:** 10.1016/j.pt.2016.09.004

**Published:** 2017-03

**Authors:** Hannah C. Slater, Lucy C. Okell, Azra C. Ghani

**Affiliations:** 1 MRC Centre for Outbreak Analysis & Modelling, Department of Infectious Disease Epidemiology, Imperial College London, UK

**Keywords:** malaria, *Plasmodium falciparum*, *Plasmodium vivax*, mathematical modelling, drug development, drug-based strategies

## Abstract

Mathematical models of the dynamics of a drug within the host are now frequently used to guide drug development. These generally focus on assessing the efficacy and duration of response to guide patient therapy. Increasingly, antimalarial drugs are used at the population level, to clear infections, provide chemoprevention, and to reduce onward transmission of infection. However, there is less clarity on the extent to which different drug properties are important for these different uses. In addition, the emergence of drug resistance poses new threats to longer-term use and highlights the need for rational drug development. Here, we argue that integrating within-host pharmacokinetic and pharmacodynamic (PK/PD) models with mathematical models for the population-level transmission of malaria is key to guiding optimal drug design to aid malaria elimination.

## Trends

Antimalarial drugs are being used in many different contexts beyond treatment of disease – increasingly with the aim of reducing malaria transmission in a community.

Each drug has different attributes – killing efficacy against asexual parasites, duration of effect, gametocytocidal activity, mosquitocidal activity, liver-stage activity (for *Plasmodium vivax*), dosing schedule and toxicity.

Drug attributes need to be rationally combined to match their usage aims based on a quantitative understanding of their properties.

For transmission reductions, the individual patient approach is less relevant and a population-level perspective is critical.

Rational approaches to combining drugs with other forms of malaria control to reduce malaria transmission can only be made using transmission models informed by field data, given the difficulty of testing all combinations of interventions in all settings.

## Extended Role of Drugs for Malaria Control and Elimination

Over the past 15 years, declines in malaria transmission have been witnessed across many malaria-endemic countries, with a 37% fall in case incidence and 60% drop in mortality rates between 2000 and 2015 [1]. Whilst much of the progress to date has been attributed to enhanced vector control [2], there has recently been increased interest in the role that drug-based strategies that extend beyond first-line treatment of malaria cases could play in further reducing morbidity and mortality and in moving towards malaria elimination. This includes the role of enhanced case-finding, wider **chemoprevention** (see Glossary) strategies to protect high-risk groups and mass treatment strategies to clear the **infectious reservoir** [3]. However, at the same time the emergence of resistance to **artemisinin** drugs and to **artemisinin combination therapies (ACT)** 4, 5, 6 (as well as to other components of the malaria arsenal such as insecticides) has demonstrated the potential fragility of current strategies. Here we argue that transmission modelling can provide a unique population-level perspective to guide the development of new antimalarial drugs to ensure that they are tailored for specific usage scenarios.

## Antimalarial Drug Properties and Malaria Transmission

The objective and purpose of antimalarial drugs, in common with other anti-infectives, has traditionally been to clear infection in an individual. Thus, during the drug development process, the central focus is on the ability of the compound to kill the parasite during the blood stage of infection or on providing chemoprevention to high-risk groups 7, 8, 9.

Mathematical models are frequently employed at this stage to guide product development 10, 11. These models incorporate two factors. The first is the **pharmacokinetics** of the compound: how the drug concentration increases and decays over time as determined by its absorption, distribution, metabolism, and excretion. This is typically described by a set of differential equations, broadly representing the physical compartments where these different effects take place. The second is the **pharmacodynamics**, describing the relationship between the drug concentration and its killing efficacy. This is summarised as a ‘dose response curve’ showing the efficacy as a function of the measured concentration in the blood. By combining these two models, predictions can be made of the likely efficacy and its decay over time for different dosing schedules. Such approaches are increasingly being evaluated to inform antimalarial drug development, including optimising the dosing schedule 12, 13, 14, 15, and to explore the impact of combinations of therapies 16, 17, 18, 19.

A key property of the current artemisinin-based therapies is their additional effect on the **gametocyte** stage of the parasite, which is responsible for onward transmission to the mosquito 20, 21. Once patent blood-stage infection is established, gametocytes develop from **asexual parasites** with the delay due to maturation of approximately 10 days in *Plasmodium falciparum* infection, and hence, following treatment, if the drug has no gametocytocidal activity, an individual may remain infectious for 20–50 days 22, 23. The gametocytocidal activity of compounds is known to differ, with most current artemisinin compounds having high efficacy against the early stages and thus reducing the post-treatment infectious period to between 10 and 20 days 22, 24, 25. However, some compounds, including **primaquine** and **tafenoquine**, attack the later gametocyte stages which reduces the post-treatment infectious period to 0–10 days 26, 27, 28. Whilst such properties were historically not a focus of the drug development agenda, their potential for clearing infection has more recently been recognised, and there is an active research agenda into the potential utility of drugs with higher levels of gametocytocidal activity 29, 30, 31.

In *Plasmodium vivax*, there is no lag between blood-stage infection and the development of gametocytes. However, treatment for *P. vivax* differs to that for *P. falciparum* due to the presence of a **hypnozoite** reservoir in the liver, which is not affected by ACTs. One class of drugs – the 8-aminoquinolines – is effective against this form, with primaquine (given as a 14-day full-dose course in contrast to its use at single low-dose for gametocyte clearance in *P. falciparum* infection) currently the only drug available from this class for treatment [8]. The ability to clear this reservoir of dormant infection has benefits for both the individual (in preventing **relapses** of disease) and for population-level transmission.

Whilst the efficacy of any antimalarial against asexual parasites and gametocytes (and for *P. vivax*, hypnozoites) determines the clearance rate of infection and onward infectivity in the individual, the relative value of these different drug actions at the population level can only be assessed by considering who in the population is transmitting infection. This **infectious reservoir** combines both those who present with clinical disease (and hence who may potentially seek treatment) and asymptomatic carriers of infection 32, 33, 34, 35.

**Figure 1:**
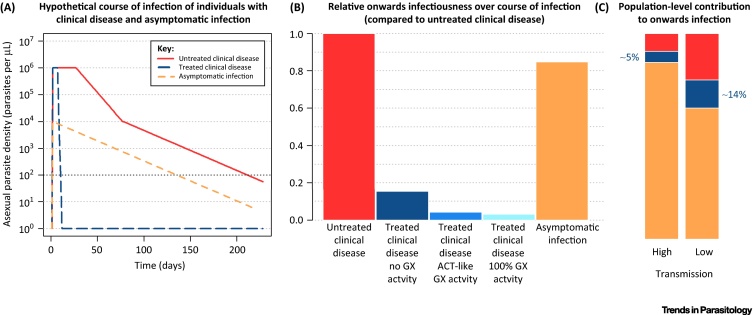
Schematic of the Relationship between an Individual's Course of Infection, Their Contribution to the Population Infectious Reservoir and the Impact of Treatment. Panel (A) shows a hypothetical typical course of infection for untreated and treated clinical disease (i.e., symptomatic infection) and asymptomatic infection. The black horizontal line indicates the limit of detection for microscopy (200 parasites per μL). Panel (B) combines the duration of infection with the infectivity to mosquitoes to produce a relative measure of onwards infectiousness for individuals with different types of infection and treatment based on parameter values presented in [88]. We assume individuals with untreated clinical disease are highly infectious for 25 days, followed by a period of 200 days where they have patent asymptomatic infection, then 100 days where they have subpatent asymptomatic infection. During these periods they are 65% and 9% as infectious as at their peak, respectively. Individuals with clinical disease who are treated with a non-artemisinin combination therapy (ACT) with no gametocytocidal (GX) activity (dark blue) are assumed to remain highly infectious for 25 days after treatment, whereas treatment with an ACT (medium blue) reduces the total duration to 10 days and the infectiousness after treatment to 9% of the pretreatment amount 22, 40, 88. A drug with perfect GX activity (light blue) renders individuals instantly noninfectious after treatment 22, 24, 25. Panel (C) considers the population-level contribution of untreated (red) and treated (dark blue) symptomatic and asymptomatic (orange) individuals based on the individual-level durations of infection presented in (A) and the relative onwards infectiousness presented in (B). We assume that 80% of individuals with clinical disease are treated, and that 30% of new infections are symptomatic in a high transmission setting and 60% in a low transmission setting [88]. Converting individual-level infectiousness to the population level allows us to see that treated individuals only contribute 5% or 14% (high and low transmission settings respectively) to the total infectiousness of a population. This indicates that improving the gametocytocidal activity of an antimalarial drug used for treatment of symptomatic cases can only potentially impact a small proportion of the total infectiousness of the population.

Figure 1 shows a hypothetical typical course of infection in these individuals and the likely reduction in onward infectivity at the population level from increasing gametocytocidal activity. It is clear from this simple schematic that clearance of asexual parasites has the greatest effect in reducing onward infectivity (as by killing asexual parasites, fewer gametocytes develop), whilst the additional benefits of gametocytocidal activity may be modest. A number of mathematical models have quantified this effect in different transmission settings 23, 36, 37, 38, 39. Despite using very different model frameworks, there is clear consensus that artemisinin-based therapies have some advantage over non-artemisinin based therapies in reducing onward transmission, in particular in areas in which a high proportion of infections seek care 23, 40. Furthermore, there is clear consensus that additional gametocytocidal activity, as provided for example by low-dose primaquine, will have negligible additional impact 23, 36, 38, 39. This is in contrast to the results obtained using an individual-patient approach 28, 41, and highlights the need to use models to evaluate impact at the population level.

A promising new avenue of research for antimalarial compounds is **mosquitocidal** activity. This has been stimulated by the recent identification of the potential for **ivermectin** (one of a number of **endectocide** compounds) to reduce the lifespan of a mosquito which ingests the compound from the blood of a treated individual. This can have two effects; first, it could reduce the probability that parasites ingested during the blood meal reach their infectious **sporozoite** stage, and secondly, it can act directly as vector control by reducing the ***Anopheles*** population. Whilst initially, focus was on the first effect, mathematical modelling has demonstrated that, at a population level, the second effect is likely to be dominant 39, 42. Thus, perhaps for the first time in antimalarial drug development, modelling is being integrated into the wider research agenda to inform the evaluation of ivermectin as a tool to aid malaria elimination [43].

## Expanding the Use of Antimalarial Drugs

The primary use of antimalarial drugs is the treatment of symptomatic cases. For this purpose, the most important properties are effective clearance of parasites and quick alleviation of symptoms. However, by taking a population perspective, the relative importance of various drug properties in different settings can be disentangled (Table 1, Key Table).

In moderate to high transmission settings, modelling has demonstrated that drugs with longer durations can have important additional benefit for the individual by providing a period of **prophylaxis** and hence reducing rates of reinfection 40, 44, 45, 46. By contrast, the gametocytocidal and mosquitocidal effects are predicted to be limited 23, 36, 37, 38, 39. This is because the proportion of the total infectious reservoir that is being treated is very small – the majority of onward transmission will be from asymptomatic (yet infectious) individuals who are not receiving treatment (Figure 1). By contrast, in a low-transmission setting, individuals with clinical disease seeking treatment will constitute a larger proportion of the infectious reservoir (Figure 1). In these settings gametocytocidal and mosquitocidal effects will have a greater proportionate impact. Furthermore, the duration of prophylaxis will become less important as transmission declines since the risk of reinfection also becomes low. For *P. vivax*, modelling has shown that, in all settings, treatments that attack the hypnozoites are critical to reduce transmission of the parasite at the population level 47, 48, 49, 50.

Drugs are also used for **chemoprophylaxis** (for visiting travellers) and chemoprevention (for those residing in endemic areas). In both scenarios, the aim is to provide protection against infection for short periods of time to at-risk groups. **Seasonal malaria chemoprevention** (SMC) was recommended by the WHO in 2012 as a 3-month course of drugs given to children under 5 years in areas with highly seasonal transmission (the Sahel region of Africa) (*http://www.who.int/malaria/publications/atoz/who_smc_policy_recommendation/en/*). A key property of the drug is its ability to prevent infection and, for this use, modelling has demonstrated the superiority of long-acting combinations [44]. One concern about SMC has been the potential delay of the acquisition of immunity and hence a shifting of malaria cases to older ages which could potentially result in an increase in total cases or increased severity of disease [51]. Whilst this has been difficult to assess in individuals, the population effect has been suggested, by modelling studies, to be potentially large 52, 53, 54.

Chemoprevention is also recommended in pregnant women residing in endemic areas, through **intermittent preventive treatment in pregnancy (IPTp)**. Here the aim is to prevent sequestration of the parasite in the placenta by clearing infection in both the placenta and circulating blood, and hence reduce morbidity in the mother and baby [55]. Whilst the focus has traditionally been on the second and third trimesters, modelling has demonstrated that the highest risk is the presence of infections towards the end of the first trimester 56, 57. Recent results have demonstrated the safety of artemisinin drugs during this period [58].

In the past 5 years, there has been increasing interest in the use of community-based administration of drugs as a means to clear the **parasite reservoir**. Typically referred to as **mass drug administration (MDA)**, this involves giving antimalarial drugs to the whole population regardless of infection status. This strategy has recently been recommended by the WHO for a series of different use scenarios (*http://www.who.int/malaria/publications/atoz/role-of-mda-for-malaria.pdf?ua=1*): (i) in low-transmission settings to clear the parasite reservoir and hence ‘accelerate’ towards elimination; (ii) in areas with high levels of drug resistance, also with the aim of accelerating towards elimination; (iii) in a time-limited manner to respond to epidemics or in complex emergencies.

The aim of MDA is to clear parasites, reduce prevalence, and maintain these gains either to reduce incidence during the upcoming transmission season, or to achieve a level of transmission so low such that local elimination is possible. Therefore, an antimalarial suitable for MDA should not only effectively clear parasites but also suppress the resurgence in transmission commonly seen in the months following the intervention. The research community has focused on the role of gametocytocidal drugs in such strategies [30], arguing that all onward transmission needs to be interrupted. However, this argument is made from an individual perspective rather than considering the impact on the population as a whole. By contrast, modelling studies have demonstrated that drugs with a longer prophylactic period are likely to have a greater effect in reducing *P. falciparum* transmission by preventing all treated individuals from being reinfected 23, 36, 37, 38, 39. Whilst gametocytocidal activity can be beneficial, the same modelling studies have shown that this factor is less important, acting only to delay the resurgence by a few weeks. This is because the reduction in population-level onwards infectivity due to the gametocytocidal activity is small compared to the long-lasting onwards infectivity of the asymptomatic untreated individuals in the population (Figure 1). Modelling has demonstrated that an ACT with an additional mosquitocidal effect could increase the impact and sustain the reductions of an MDA in both high- and low-transmission settings [42].

For *P. vivax*, however, MDA with drugs that treat only blood-stage infection are predicted to have only a transient effect, whilst addition of a liver-stage drug is predicted to be highly effective 49, 59, 60. This is due to the large effect at a population-level of relapsing infection [61].

Fewer modelling studies have examined the role of MDA in epidemic control or complex emergencies. One study showed that MDA could play an important role in mitigating the effect that Ebola virus disease had on access to healthcare and hence treatment of malaria during the 2014–15 epidemic in West Africa [62]. In this study the duration of prophylaxis of the drug again was found to be more important than gametocytocidal activity. Another modelling study demonstrated the cost-effectiveness of provision of antimalarials to Ebola case contacts [63]. Both studies highlight the benefit of models to guide malaria control in emergency situations.

## Tackling the Emergence of Drug Resistance

Assessing the risk of drug resistance to a particular compound is embedded in the antimalarial drug development process from the preclinical phases [64]. Numerous mathematical models have been used to quantify how different drug properties could influence both the emergence and spread of resistance. A key measure is the ease with which resistance can develop in the laboratory in parasite cultures exposed to suboptimal levels of the drug. This appears to correlate to some extent with resistance in the field, with, for example, **atovaquone** resistance being easy to develop in the laboratory, and artemisinin resistance relatively harder [64]. The probability of a particular combination of mutations occurring has been incorporated into mathematical models, taking into account global numbers of cases to estimate a time until a resistant strain begins to establish and spread somewhere in the world [65].

**Figure 2:**
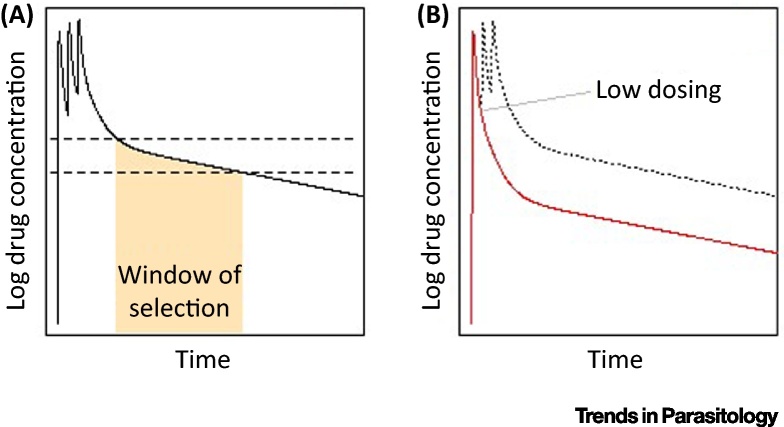
Pharmacokinetics and Vulnerable Time-Points for Selection of Partial Resistance. (A) During drug elimination, the unbroken line shows the drug concentration in the blood of a patient who takes the full course of a three-dose drug regimen, which has a long elimination half-life and so its concentration wanes gradually over time. This schematic is based on the antimalarial piperaquine. The window of selection is the time during which drug concentrations are sufficiently high to allow partially resistant parasites to survive, but kill sensitive parasites (in between the horizontal broken lines). During this period, this selection will usually act on parasites from new infections. Above these concentrations (above the upper broken line), both sensitive and partially resistant parasites are killed by the drug, and below these concentrations (below the lower broken line), both sensitive and partially resistant parasites can survive, so there is no selection. The window of selection would be longer for highly resistant parasites compared with parasites with a low level of partial resistance. (B) The red line shows the drug concentration in the blood of a patient who receives a lower than recommended amount of the drug, in this case because they take only one dose instead of three. Drug concentration therefore does not remain at a high enough level for a sufficient length of time to kill all parasites in the initial infection, potentially selecting for partially resistant parasites.

The PK/PD profile of an antimalarial is another key determinant of the development of resistance. Drugs with long half-lives are present in patients at suboptimal concentrations for a period of time, and therefore there can be selection for partially resistant parasites when a patient is exposed to new infections after being initially treated (Figure 2A). Models can be used to approximately quantify the length of this window of selection 66, 67, 68, 69, 70. Partial resistance is often an important step for the parasite on the pathway to higher grade resistance – for example, the evolution of resistance to **sulphadoxine-pyrimethamine** (**SP**) – and can rapidly reduce the duration of protection against reinfection. This reduction can be estimated using models where direct clinical data are lacking [71], and is important for prophylactic interventions such as SMC. However, some model outputs have highlighted the difficulties of using laboratory measures to directly parameterise models of treatment in humans. For example, it has been shown that estimates of the **IC50** of several ACT partner drugs from the laboratory were very different from those estimated from a within-host PK/PD model [72]. A review has suggested ways in which such models could be further developed to improve their utility for drug development, for example, by simultaneously including human immunity, parasite dynamics, stage-specificity of drug action, and by fitting to PK/PD data [15].

Another important source of selection pressure for partial resistance is low dosing (Figure 2B), which has been explored using mathematical models [73]. Suboptimal dosing can occur when patients do not fully adhere to the treatment regimen, and therefore creating simple and short-dose regimens is important. Synthetic artemisinins which are currently in development have been designed to have longer half-lives, and therefore patients will likely require fewer than the three doses of artemisinin currently given as part of most ACTs [74]. Suboptimal dosing can also occur even when the patient takes the recommended dose, due sometimes to the same dose of drug being prescribed for broad weight bands, or different metabolism of the drug in different age groups 14, 75. Modelling, based on pharmacokinetic data, can be used to explore alternative dosing guidelines and optimise drug concentrations across age and weight groups 13, 16. One such analysis suggests that twice-daily rather than once-daily ACT doses could improve drug efficacy whilst keeping drug concentrations below potentially toxic levels [76].

Modelling can also explore hypotheses about how resistance evolves and spreads. For example, given the observed mutation rate of *P. falciparum*, it was initially assumed that drug resistance to **chloroquine** and SP had evolved many times independently. However, genetic data showed that high-grade resistance had in fact evolved only a very small number of times, and then spread. Mathematical modelling was able to reconcile these observations by quantifying bottlenecks for resistant strains, not only during its initial evolution but then during onward transmission [77]. A within-host model also helped to elucidate that artemisinin resistance was due to the **ring stages** of the parasite becoming insensitive to the drug, before this was confirmed in the laboratory [78]. Furthermore, novel hypotheses have been generated about whether using the minimum drug dose required to be clinically effective could actually prevent the development of high-grade resistance [79], though this is contradicted by results from other models [12].

A major advance in combating antimalarial drug resistance was made in the development and adoption of artemisinin combination therapies 80, 81. The large reduction in the probability of resistance developing when using two drugs simultaneously, relative to monotherapies, has been quantified in mathematical models 70, 80, 81. However, the reduced killing of artemisinin-resistant parasites by artemisinin derivatives increases the exposure of parasites to partner drugs, and indeed piperaquine resistance has already been rapidly selected in areas of Cambodia with artemisinin resistance [4]. Further advances in this field are ongoing, with trials of triple combination therapy underway in Cambodia (*http://www.wwarn.org/working-together/partner-projects/tracking-resistance-artemisinin-collaboration*). Outputs from models had highlighted a potential problem of mismatched PK/PD profiles in existing ACTs, where the longer-acting partner drugs remain in the blood after the shorter-acting artemisinin derivatives were cleared from the patient [67]. The current trials have therefore created triple therapies consisting of an artemisinin derivative, plus either **mefloquine** and **piperaquine**, which are active for a month or more, or **lumefantrine** and **amodiaquine**, which both have a prophylactic period of ∼2 weeks [46]. The triple combination design also harnesses an intriguing finding that a mutation conferring resistance to one partner drug appears to confer sensitivity to the other partner drug 82, 83. Modelling the impact of these triple combinations on resistance development is an important area for future research. It has also been proposed that adding primaquine to ACT could impede the spread of resistance, although modelling found a relatively small benefit of such a strategy [37]. Another proposed strategy for resistance management is use of **multiple first line therapy (MFT)** in populations. The results from different models suggest different degrees of advantage or disadvantage of MFT over the current strategy of sequentially replacing drugs when resistance develops 65, 84, 85, 86. For such questions, further modelling has a key role to play, as it is difficult to envisage how the hypothesis could ever be tested in the field.

## Future Role of Models to Guide Drug Development

There has been a rapid growth in the development and application of models to guide drug development for antimalarials, including both PK/PD modelling to identify appropriate candidates for development, and transmission models to guide drug deployment and use scenarios. As outlined here, models are now being used to guide development of new compounds, to improve dosing and adherence of existing compounds, and to inform appropriate deployment strategies at the individual and population levels.

Despite this progress, there remains a lack of the population-level insight that can be obtained from modelling embedded in the current research agenda for drug-based strategies. This is perhaps best demonstrated by the example of the development of the research agenda for low-dose primaquine. Despite consistent outputs across a range of modelling approaches demonstrating the very small potential impact of adding this to existing ACTs, this agenda has been vigorously pursued and many countries are now adopting this into policy [87]. Earlier integration of modelling approaches would therefore be beneficial.

A key area for further research is to integrate PK/PD and transmission modelling approaches (see Outstanding Questions and Box 1). This should involve the integration of population-level modelling in *in vitro* laboratory assessment of drug candidates as well as better characterisation of PK/PD effects within the population-level models. Such an approach could enable a better understanding of the potential role of new candidate drugs, alone and in combination, as well as guide potential alternative deployment strategies.

Finally, with the ever-present threat of the spread of artemisinin- and ACT-resistance, there is a pressing need to consider population-based strategies to reduce the spread of existing resistant parasites and to delay the emergence of resistance to new compounds. Given the inherent difficulty in testing such strategies in the field, modelling will likely remain the only route through which strategies such as MFT can be evaluated. This will require a better understanding of the relationship between laboratory measures of resistance and PKPD in humans, and epidemiological spread of resistance.

Outstanding Questions How can model predictions be tested empirically? To what extent can evidence from programmatic deployment be used to test and validate transmission and PK/PD models?

How can modelling link insights from the laboratory to potential in the field? How can quantitative measures of individual drugs be used to improve the quality of predictions?

How do drug interactions within the host (PK/PD) affect population transmission? Can modelling better inform the choice of drug combinations?

How does adherence affect population transmission, and what are the appropriate dosing strategies to counter poor adherence? Does this differ for different usage scenarios?

What drug properties and combinations are best suited for active case finding in low-transmission settings and approaching elimination?

Can drug-based strategies be used to prevent resurgence/outbreaks? How rapidly do these need to be deployed?

What drug properties and deployment strategies are needed to ensure elimination of *Plasmodium vivax*?

What strategies (drug properties, combinations, and deployment) can be employed to slow the emergence and spread of drug resistance?

## Figures and Tables

**Figure I:**
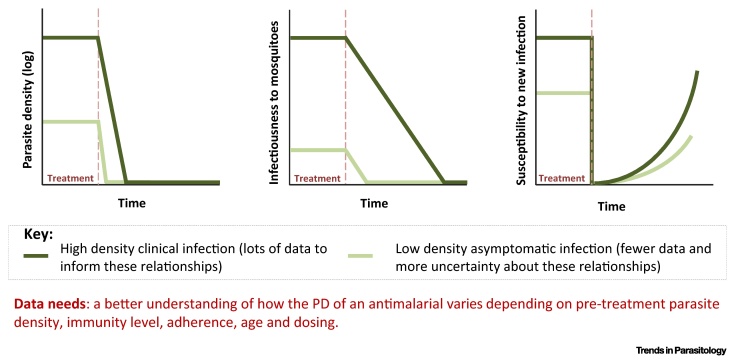
Hypothetical Pharmacodynamics (PD) in Individuals with High- and Low-density Malaria Infections.

**Table 1:** Key Table Summary of the Relative Importance of the Different Drug Properties for the Different Use Scenarios^a^

	Property					Refs
	Clearance of Blood-Stage Parasites	Duration of Prophylaxis	Enhanced Gametocytocidal Activity^b^	Mosquitocidal Activity	Hypnozoite Activity^c^	
First-line treatment	+++	++	+	+	++	23, 25, 42, 45, 46, 49, 50, 61
Chemoprevention: children	+++	+++	??	??	??	44, 52, 89, 90
Chemoprevention: pregnant women	+++	+++	??	??	??	56, 57
MDA: elimination	++	++	+	+++	+++	36, 37, 38, 39, 42, 60
MDA: emergencies	++	++	+	??	??	62, 63

a The rankings are based on a review of the modelling literature with the key references shown in the final column. +++ = very important; ++ = moderately important; + = limited importance; ?? = further research required.

b Enhanced gametocytocidal activity refers to that greater than current ACTs.

c Relevant to *Plasmodium vivax* only.

## Box 1 Integrating PK/PD and Transmission Modelling Approaches

The PK/PD of antimalarials is typically well characterised in individuals with clinical infection, but less so in asymptomatic infections that also contribute to onward infection. Information on both is required for assessment of drug candidates for drug-based strategies that target non-symptomatic individuals such as SMC and MDA, as shown in Figure I. Once characterised, these can be directly incorporated in transmission models to estimate the impact in populations with varying levels of clinical disease and asymptomatic infection.

## Glossary

Amodiaquine: partner drug for first-line treatment; also used as part of recommended regimen for seasonal malaria chemoprevention (SMC).
*Anopheles*: species of mosquito responsible for transmission of the malaria parasite.
Artemisinin: a class of drugs, isolated from the plant *Artemisa annua*, which provide rapid action against *P. falciparum* infection.
Artemisinin combination therapy (ACT): a combination of an artemisinin derivative with a longer-acting antimalarial drug that has a different mode of action.
Asexual parasite: collective term for the parasite forms during the blood-stage infection that invade the red blood cells and distinguish from the infectious form (gametocyte).
Atovaquone: partner drug for first-line treatment; also used for chemoprophylaxis.
Chemoprevention: intermittent administration of a full treatment course of an antimalarial medicine to prevent malarial illness.
Chemoprophylaxis: administration of a medicine, at predefined intervals, to prevent either the development of an infection or progression of an infection to manifest disease.
Chloroquine: early drug used for first-line treatment prior to the widespread development of resistance from the 1990s onwards.
Endectocide: an antiparasitic drug that is active against both endoparasites and ectoparasites.
Gametocyte: sexual stage of malaria parasites that can potentially infect *Anopheles* mosquitoes when ingested during a blood meal. This stage plays no role in clinical disease and hence was not initially a focus for drug developers.
Hypnozoite: persistent liver stage of *P. vivax* (and *P. ovale*) malaria that remains dormant in host hepatocytes for variable periods before activation and development into a pre-erythrocytic schizont which then causes a blood-stage infection (relapse).
IC50: the concentration of a drug that is needed to give half of its maximal biological efficacy.
Infectious reservoir: the product of the number of people harbouring parasites that can be transmitted on to mosquitoes and their relative infectiousness.
Intermittent preventive treatment in pregnancy (IPTp): a full therapeutic course of antimalarial medicine given to pregnant women at routine prenatal visits, regardless of whether the woman is infected with malaria.
Ivermectin: mosquitocidal drug currently used to treat onchocerciasis.
Lumefantrine: partner drug for first-line treatment.
Mass drug administration: administration of antimalarial treatment to every member of a defined population or living in a defined geographical area (except those for whom the medicine is contraindicated) at approximately the same time and often at repeated intervals.
Mefloquine: partner drug for first-line treatment; also used for chemoprophylaxis.
Mosquitocidal: a drug or compound that kills mosquitoes.
Multiple first-line therapy (MFT): a policy to administer more than one antimalarial combination for first-line treatment at a population level; this can be geographically stratified or individually randomised.
Parasite reservoir: people harbouring parasites, regardless of their onward infectivity.
Partner drug: a longer-acting antimalarial drug given in combination with an artemisinin drug that has a different mode of action.
Pharmacodynamics: the biochemical and physiological effects of drugs and their mechanism of action.
Pharmacokinetics: the absorption and distribution of drugs through the body.
Piperaquine: partner drug for first-line treatment; used for prophylaxis in China and India prior to the development of resistance.
Primaquine: the only licensed drug for treatment *of Plasmodium vivax* hypnozoites. Also recommended at low-dose for clearance of gametocytes in *Plasmodium falciparum* infections in areas with low transmission.
Prophylaxis: any method of protection from or prevention of disease.
Relapse infection: recurrence of asexual parasitaemia in *P. vivax* or *P. ovale* infections arising from hypnozoites.
Ring stage: young, usually ring-shaped malaria trophozoites growing within host red blood cells, before pigment is evident by microscopy.
Seasonal malaria chemoprevention (SMC): intermittent administration of full treatment courses of an antimalarial medicine during the malaria season to prevent malarial illness.
Sporozoite: motile stage of the malaria parasite that is inoculated by a feeding female anopheline mosquito and may cause infection.
Sulphadoxine-pyrimethamine (SP): previously first-line therapy prior to widespread development of resistance. Used as part of the recommended regiment for seasonal malaria chemoprevention (SMC) and for intermittent preventative treatment during pregnancy (IPTp).
Tafenoquine: drug in development for the treatment of *P. vivax.*
Vector control: measures of any kind against malaria-transmitting mosquitoes intended to limit their ability to transmit the disease.
